# Detection of emerging HoBi-like *Pestivirus* (BVD-3) during an epidemiological investigation of bovine viral diarrhea virus in Xinjiang: a first-of-its-kind report

**DOI:** 10.3389/fmicb.2023.1222292

**Published:** 2023-07-04

**Authors:** Ningning Yang, Mingguo Xu, Zhenguo Ma, Honghuan Li, Shengnan Song, Xiaoxiao Gu, Jingnan Liu, Zhonglian Yang, Hongji Zhu, Hailong Ma, Jihai Yi, Yong Wang, Zhen Wang, Jinliang Sheng, Chuangfu Chen

**Affiliations:** ^1^College of Animal Science and Technology, Shihezi University, Shihezi, China; ^2^Key Laboratory of Control and Prevention of Animal Disease, Xinjiang Production and Construction Corps, Shihezi, China; ^3^Co-Innovation Center for Zoonotic Infectious Diseases in the Western Region, Shihezi, China

**Keywords:** HoBi-like Pestivirus, bovine viral diarrhea virus, cattle, phylogenetic analysis, genotype, Xinjiang

## Abstract

Xinjiang pastoral area is the second largest pastoral area in China, accounting for 26.8% of the available grassland area in the country, and the geographical advantage of cattle breeding industry is very obvious. Bovine viral diarrhea virus (BVDV) has always been one of the important viral diseases that have plagued the development of cattle farming industry in the world. As one of the main pastoral areas of China’s cattle farming industry, the Xinjiang pastoral area has also been deeply affected. In this study, 6,153 bovine serum samples were collected from 18 large-scale cattle farms in 13 cities in Xinjiang. The antibodies and antigens of 6,153 and 588 serum samples were detected by serological detection methods, respectively. Ten serum samples, which were antigen-positive by ELISA, were randomly selected for RT-PCR detection, sequencing, and phylogenetic analysis of suspected HoBi-like *Pestivirus* (HoBiPeV) strains. The results showed that the positive rates of BVDV antibodies and antigens were 53.68% (3,303/6,153) and 6.12% (36/588), respectively. One of the 10 randomly selected seropositive samples was infected with the HoBiPeV strain. HoBiPeV, also referred to as BVDV-3, is an emerging atypical *Pestivirus* that occurs in cattle and small ruminants, and its clinical signs are similar to those of BVDV infection. Based on the whole genome of the BVDV-3 reference strain (JS12/01) on the GenBank, the homology of the detected strain was 96.02%. The whole genome nucleotide sequence was submitted to the GenBank database, and the gene accession number was obtained: OP210314. The whole genome of isolate OP210314 was 12.239 nucleotides and contained a 5′-UTR of 340 nucleotides, a 3′-UTR of 199 nucleotides, and a large open reading frame (ORF) encoding a polyprotein consisting of 3,899 amino acids. In conclusion, the prevalence rate of BVDV infection in Xinjiang dairy cows is high, and the genetic diversity is increasing. This study successfully identified and isolated HoBiPeV in Xinjiang for the first time, posing a potential threat to the cattle industry in Xinjiang.

## Introduction

Bovine viral diarrhea/mucosal disease (BVD/MD) is an infectious disease caused by the bovine viral diarrhea virus (BVDV; Richter et al., 2017; Deng et al., 2020). BVDV can infect various animals, such as cattle, pigs, sheep, and deer (Krametter-Froetscher et al., 2010; Blome et al., 2017; Chen et al., 2021; Zhang et al., 2022). BVDV primarily infects cattle in its natural environment, with calves being the most vulnerable and susceptible hosts. Animals infected with BVDV can experience symptoms such as fever, diarrhea, respiratory symptoms, mucosal erosion, and pregnant animals may experience abortion, stillbirth, or abnormal fetuses (Baker, 1995; Blome et al., 2017; Zhou et al., 2022). The World Organization for Animal Health (WOAH) classifies BVDV as a Class III infectious disease, according to the degree of harm to animal health and public health.

BVDV is a member of the *Flaviviridae* family, belonging to the *Pestivirus* genus together with classical swine fever virus (CSFV) and border disease virus (BDV; Chen et al., 2021; Yang et al., 2022). According to the recommendations of the *Flaviviridae* Study Group of the International Committee on Taxonomy of Viruses (ICTV), BVDV can be divided into three genotypes: BVDV-1 (*Pestivirus* A), BVDV-2 (*Pestivirus* B), and atypical *Pestivirus* or BVDV-3 (*Pestivirus* H; Smith et al., 2017). BVDV-1 can be further divided into at least 23 subtypes (1a-1w), BVDV-2 can be further divided into four subtypes (2a–2d), and BVDV-3 can be divided into Brazilian, Thai, Italian, and other sources (Smith et al., 2017; Yeşilbağ et al., 2017; Deng et al., 2020). Among them, BVDV-1 strains are the most prevalent worldwide and often used in laboratory study and vaccine development. BVDV can be divided into cytopathic (CP) and non-cytopathic (NCP) types, depending on whether they can cause cytopathic effect (CPE) on host cells, and both biotypes are pathogenic. However, only NCP strains can cause persistent infection (PI) in cattle (Lackner et al., 2004; Yang et al., 2022). The existence of PI cattle is also one of the major reasons why BVDV is difficult to prevent and eliminate.

BVDV was initially discovered in 1946 in cattle exhibiting symptoms such as peptic ulcers and dysentery (Olafson et al., 1946). It was successfully isolated in 1957 (Lee and Gillespie, 1957). In 1980, BVDV was first isolated in China, confirming the virus presence in the country (Li et al., 1983). From 1980 to 2013, BVDV serological investigations conducted by prominent researchers including Zheng et al. (1991), Wang et al. (1996), Qiu et al. (1998, 2000), and Shang et al. (2013) revealed that BVDV infection was prevalent in most regions of China, such as Inner Mongolia, Shaanxi, Gansu, Ningxia, Qinghai, Xinjiang, Heilongjiang, Liaoning, Jilin and Henan, where cows exhibited the highest antibody positive rate. Nevertheless, there were notable disparities in the infection situation among different regions or farms within the same region.

Xinjiang boasts vast natural grasslands, with approximately 57 million hectares of usable natural grasslands and a diverse range of livestock (Xinjiang Geophysical Network, 2014). It is one of the four major pastoral areas in China. Cattle breeding in Xinjiang have unique geographical advantages, and the proportion of cattle in the total livestock species population is second only to sheep, ranking second in Xinjiang (Chao et al., 2016; Zhou, 2018). With the rapid development of animal husbandry in Xinjiang, the traditional free-range culturing mode has given way to a large-scale, intensive, and healthy culturing mode. The scale of farming is expanding, and the number of farms is increasing. However, with the continuous expansion of the breeding scale, various epidemics have emerged, and among them, BVDV is one of the significant diseases that seriously affect the healthy development of the cattle industry in Xinjiang.

In recent years, the survey data demonstrate a severe BVDV infection in Xinjiang. Guo (2007) conducted an epidemiological survey of BVDV in the northern region of Xinjiang and found that the average prevalence of BVDV was 35.40%, and all the samples collected were BVDV-1 type. Li et al. (2009) found a nucleic acid positive rate of 39.06% in the epidemiological survey of BVDV in the Shihezi region, and identified the prevalent strain as the BVDV-1b subtype. In the period from 2010 to 2012, our research team conducted an epidemiological survey on BVDV from seven areas including Shihezi, Manas, Kashgar and Aksu, and found that the average prevalence rate of BVDV was 18.61%, and found that the prevalent strain was mainly BVDV-1b subtype (Ren, 2010). Wang et al. (2015) carried out serological detection of BVDV on cattle farms in Xinjiang, and isolated 17 strains of BVDV-I and 4 strains of BVDV-II. In the period from 2016 to 2018, our research team conducted an epidemiological survey of BVDV in cattle farms in some areas of Xinjiang, and found that BVDV is widespread in Xinjiang, among which 1q strain is the most prevalent (He et al., 2020). In summary, these survey results indicate that there is a serious phenomenon of BVDV infection in Xinjiang’s cattle farming industry, and the epidemic types of the virus are diverse, which has brought serious economic losses to Xinjiang’s animal husbandry. However, the specific digital economic losses still need further investigation and research. However, it has been reported that the average annual production loss caused by BVDV infection is €42.14–€67.19 per animal (Pinior et al., 2019). In Australia, the economic loss caused by BVDV can reach up to AUD 50.9 million annually (McGowan et al., 2020).

The prevention and control of BVDV is crucial for the cattle industry, and vaccination and culling of persistently infected animals are two important measures in this regard. However, due to the rapid variation of BVDV strains, an effective vaccine is not yet available. Some European countries have launched BVDV eradication programs to combat this issue, yet our country has not implemented such program (Yue et al., 2022). Therefore, it is imperative to implement long-term and continuous monitoring of BVDV in cattle to evaluate the efficacy of prevention and control methods, including vaccination and tracing the source of infection. The aim of this survey to assess the prevalence of BVDV in Xinjiang, as well as to isolate and identify any new strains that may be emerging in the region, This study is crucial for the healthy development of the cattle industry, as it will provide insights into the current status of BVDV and help develop more effective prevention and control strategies.

## Materials and methods

### Ethics statement

This study was endorsed by the Animal Experimental Ethical Committee of the First Affiliated Hospital of Medical College, Shihezi University, and procured the written informed consent of the proprietor for the participation of their livestock. The consent form explicitly delineated the research’s objectives, procedures, potential risks, and benefits. Furthermore, we meticulously adhered to the principles of animal welfare and utilized appropriate measures to minimize the distress and discomfort experienced by the animals.

### Study area and samples collection

**Figure 1:**
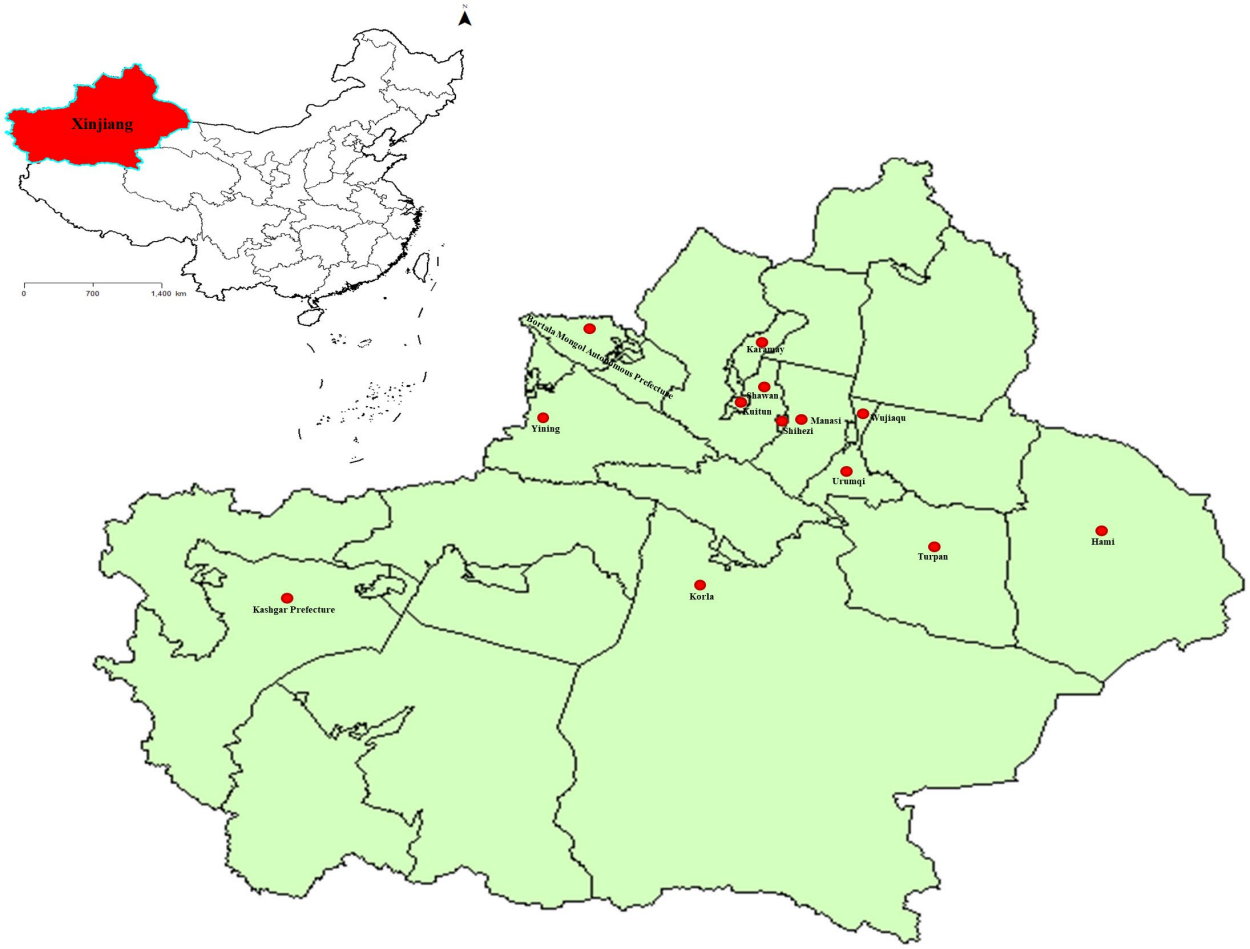
The sampling points of BVDV in Xinjiang, China. The standard map of China was obtained from the national standard map service (http://bzdt.ch.mnr.gov.cn/). The red area represents the location of Xinjiang in China, while the red dot marks the cities where the samples were collected in Xinjiang.

From 2017 to 2020, our team collected a total of 6,153 whole blood samples from 18 distinct cattle farms located across 13 cities in Xinjiang, China (Figure 1; Table 1). The collected whole blood samples were carefully transported to the Pathology Laboratory at the School of Animal Science and Technology, Shihezi University for serum separation, and were subsequently stored at a temperature of −20°C for preservation.

**Table 1:** Geographical distribution of serum samples.

Herd No.	Locations	cities	No. sample collected	Vaccine immunization	Sample source
1	Eastern region	Hami	276	No	Adult cattle
2		Turpan	546	No	Adult cattle
3	Southern region	Kashgar Shehiri	541	No	Adult cattle
4		Korla	270	No	Adult cattle
5	Westerns region	Ili Kazakh Autonomous Prefecture	401	Yes	Adult cattle
6		Ili Kazakh Autonomous Prefecture	236	Yes	Adult cattle
7		Ili Kazakh Autonomous Prefecture	84	Yes	Adult cattle
8		Jinghe	92	Yes	Adult cattle
9	Northern region	Karamay	92	No	Adult cattle
10		Kuitun	643	No	Calves
11		Shawan	68	No	Calves
12		Shawan	543	No	Calves
13		Shawan	368	No	Adult cattle
14		Shawan	461	No	Adult cattle
15		Shihezi	735	No	Adult cattle
16		Manasi	472	No	Adult cattle
17		Urumqi	92	No	Adult cattle
18		Wujiaqu	553	No	Adult cattle
Total			6,153		

### BVDV antibody and antigen detection

The collected serum samples underwent screening for both antibodies and antigens utilizing the BVDV indirect ELISA antibody test kit developed by our research team (data not yet published) and the IDEXX BVDV Ag/Serum plus Test (United States), respectively.

### RT-PCR

Ten samples showing positive antigen detected by ELISA were randomly selected and subsequently verified by RT-PCR. In brief, the viral RNA genome was extracted utilizing a biospin virus RNA extraction kit (Bioer Technology, China), and reverse transcribed to synthesize cDNA (CWBIO, China), following the manufacturer’s instructions. The primer sequences were delineated in Table 2. The amplification products underwent electrophoresis with 1% agarose gels, and were scrutinized under a gel image analysis system (Bio-Rad, United States). The purposeful bands were individually excised from the gel, recovered, and purified using a TIANgel purification kit (TIANGEN, China). The purified products were subsequently cloned into pMD-19-T vector (TaKaRa, Japan), and the positive clones (three per sample) were sequenced by Rui Biotech (Beijing, China).

**Table 2:** Primers used in this study.

Target	Forward primer (5′-3′)	Reverse primer (5′-3′)	Product size (bp)
5′-UTR of BVDV-1/2	GCTAGCCATGCCCTTAGTAG	CCATGTGCCATGTACAGC	289
5′-UTR of BVDV1/2/3	CCTAGCCATGCCCTTAGTAGGAC	GAACTCCATGTGCCATGTACA	296
A	CCCAAATTAATAATTTGGTCTAGG	ACTCTTATGTTCCAGTTTCT	1804
B	GTGTAAGAAGGGTAAGAGGTT	GAGCCACTACGATAATCACAA	1904
C	ATTCAACTGGACTGAGACGCT	TGTTTGCTCTGGCTTAG	1,485
D	TAGCCGGTGGAACCAACGT	TCCTACTCTACCCCGTCTC	2090
E	CTGAAGTGTGAGAAAAGAGTC	CTGAAGTGTGAGAAAAGAGTC	1839
F	TTGGAAATCCCTTGAGA	ACCCTTCTTGCTGGTATTGTA	1849
G	CCCTTATTTGAGGAACTGT	GAAAGCCGTCATCTCCACA	1,309
H	ATGGTGTATGCTTTTTGTGAGAG	GGCCGTTAAGGATTTTC	1,102

### Phylogenetic analysis

The nucleotide sequences were systematically aligned and scrutinized using the SeqMan tool integrated within the DNAStar software package (DNAStar Inc., United States; Deng et al., 2015; Kumar et al., 2018). Phylogenetic reconstruction of the 5’-UTR and complete genome sequences was performed using the neighbor-joining method in MEGA 7.0 software (Tamura et al., 2004). The statistical robustness of the phylogenetic tree was assessed by bootstrapping the data set with 1,000 replicates.

### Cell culture and virus isolation

Madin-Darby bovine kidney (MDBK) cells were procured from the National Collection of Authenticated (Shanghai, China) and propagated in Dulbecco’s modified Eagle’s medium (DMEM; Gibco, United States) supplemented with 10% heat-inactivated fetal bovine serum (FBS; Gibco, United States) at 37°C with 5% CO_2_. To isolate the virus (Zhu et al., 2016; Chen et al., 2021), 200 μl of BVDV-positive serum after filtration was inoculated into MDBK cells with DMEM but without FBS. After 2 h of infection, the cell culture medium was discarded, and fresh 1% FBS was supplemented. Subsequently, the virus-containing cell cultures were collected after 5 days, and subjected to three cycles of repeated freezing and thawing at −80°C. The above-described procedure was repeated with 200 μl of virus cultures inoculated into MDBK cells until passage 10 (P10), with a passage interval of 5 days. The CPE on MDBK cells were monitored under an inverted microscope (Nikon, Japan).

### Indirect immunofluorescence assay

MDBK cells were subjected to BVDV infection at a multiplicity of infection (MOI) of 10, and incubated for 36 h, serving as the experimental group (BVDV-infected MDBK group). MDBK cells cultured in DMEM were used as the control group (mock-infected MDBK group). The IFA was performed as previously described (Zhang et al., 2022). The IFA protocol was slightly modified, whereby MDBK cells were first incubated with BVDV-positive serum (diluted 1:300) as the primary antibody, followed by fluorescein isothiocyanate (FITC)-labeled rabbit anti-bovine IgG (diluted 1:500; Solarbio, China) as the secondary antibody. Finally, the cells were observed under a confocal laser scanning microscope (C2 + confocal microscope, Nikon, Japan), and images were analyzed using NIS-Elements Viewer software (Nikon, Japan).

### Statistical analysis

The statistical analysis of the data was carried out using the Chi-square test method in SPSS 17.0 (SPSS Inc., Chicago), and a *p* value <0.05 was considered to indicate statistically significant differences. GraphPad Prism 8.0 software (Graph-Pad Software Inc., United States) was employed for data visualization.

## Results

### Detection of BVDV antibody and antigen in serum samples

**Figure 2:**
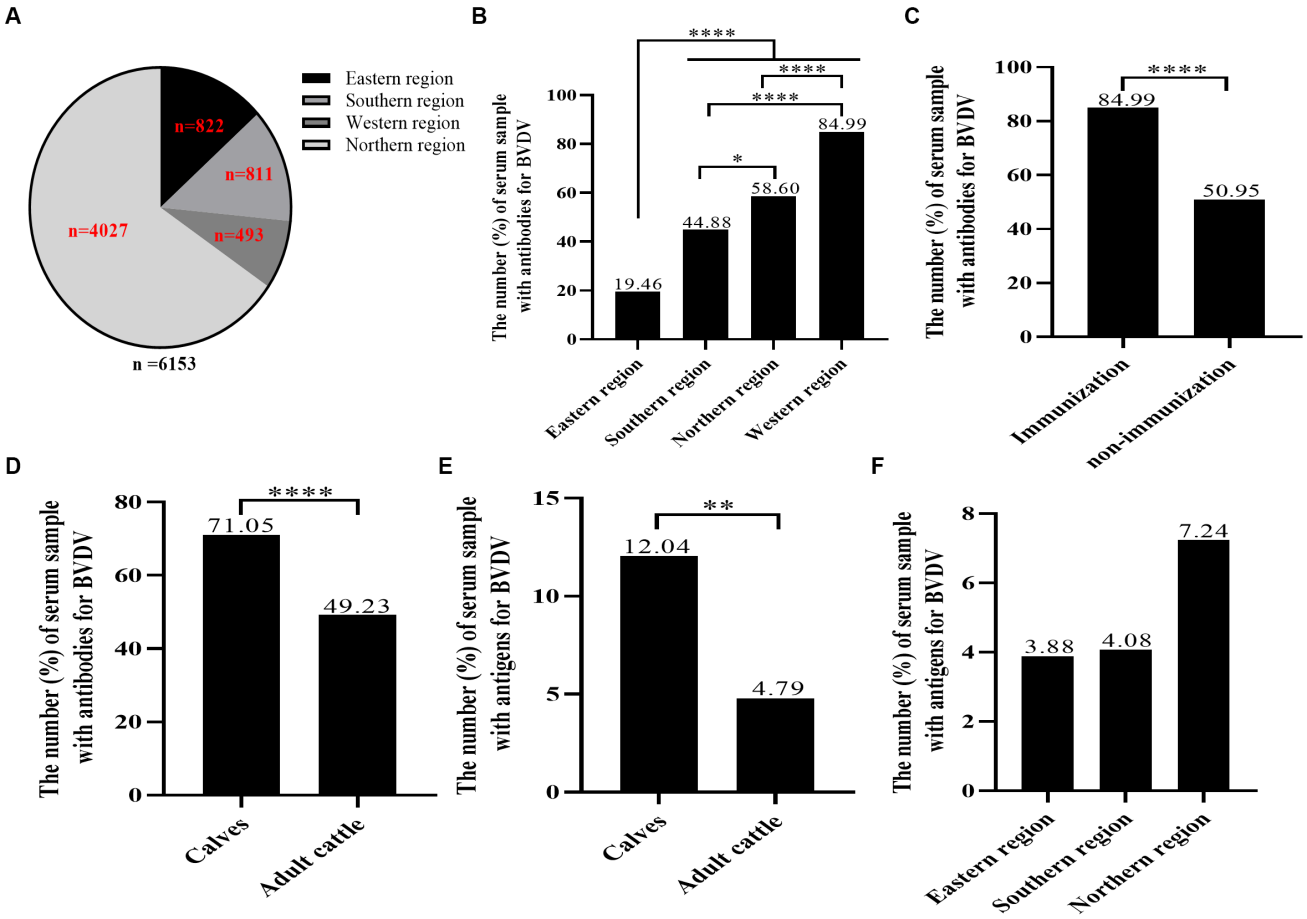
The seroprevalence of BVDV in individual animals. A total of 6,153 bovine blood samples were gathered from 18 farms, comprising calves and adult cattle, in 13 cities across four distinct regions. BVDV antibodies and antigens were identified through our research team’s BVDV indirect ELISA antibody test kit and IDEXX BVDV Ag/Serum Plus Test, respectively. **(A)** The distribution of BVDV samples by region. **(B)** The analysis of the BVDV antibody prevalence in the four regions. **(C)** The analysis of BVDV antibody positive rate of immunized cattle and non-immunized cattle. **(D,E)** The analysis of BVDV antibody and antigen levels of calves. **(F)** The analysis of BVDV antigen levels of eastern region, southern region and northern region. Statistical significance was set at *p* < 0.05 (*), *p* < 0.01 (**), and *p* < 0.0001 (****).

A total of 6,153 bovine serum samples from 18 herds spanning 13 cities in Xinjiang were systematically collected (Figures 1, 2A; Table 1). The average positive rate of BVDV-antibody in bovines was calculated to be 53.68% (3,303/6,153). It was observed that the antibody positive rate of the eastern region was comparatively lower at 19.46%, which was significantly different from the southern region (44.88%), northern region (58.60%), and western region (84.99%, Figure 2B). The antibody positive rate of the southern and northern regions was significantly lower than that of the western region. Hence, the western region had the highest antibody positive rate, possibly because the region had vaccinated their cattle with the BVDV vaccine 4 months before sampling. The antibody positive rate of immunized cattle was significantly higher than that of non-immunized cattle (Figure 2C). The antigen levels of the western region were not tested since they had already vaccinated. Interestingly, the antibody and antigen levels of calves were significantly higher than those of adult cattle (Figures 2D,E), which might be due to maternal antibody or vertical transmission. As indicated in Figure 2F, the eastern region had the lowest antigen positive rate at 3.88%, followed by the southern region (4.08%), whereas the northern region had the highest antigen positive rate of 7.24%. However, there was no significant difference in the antigen positive rate among the three regions.

### RT-PCR

**Figure 3:**
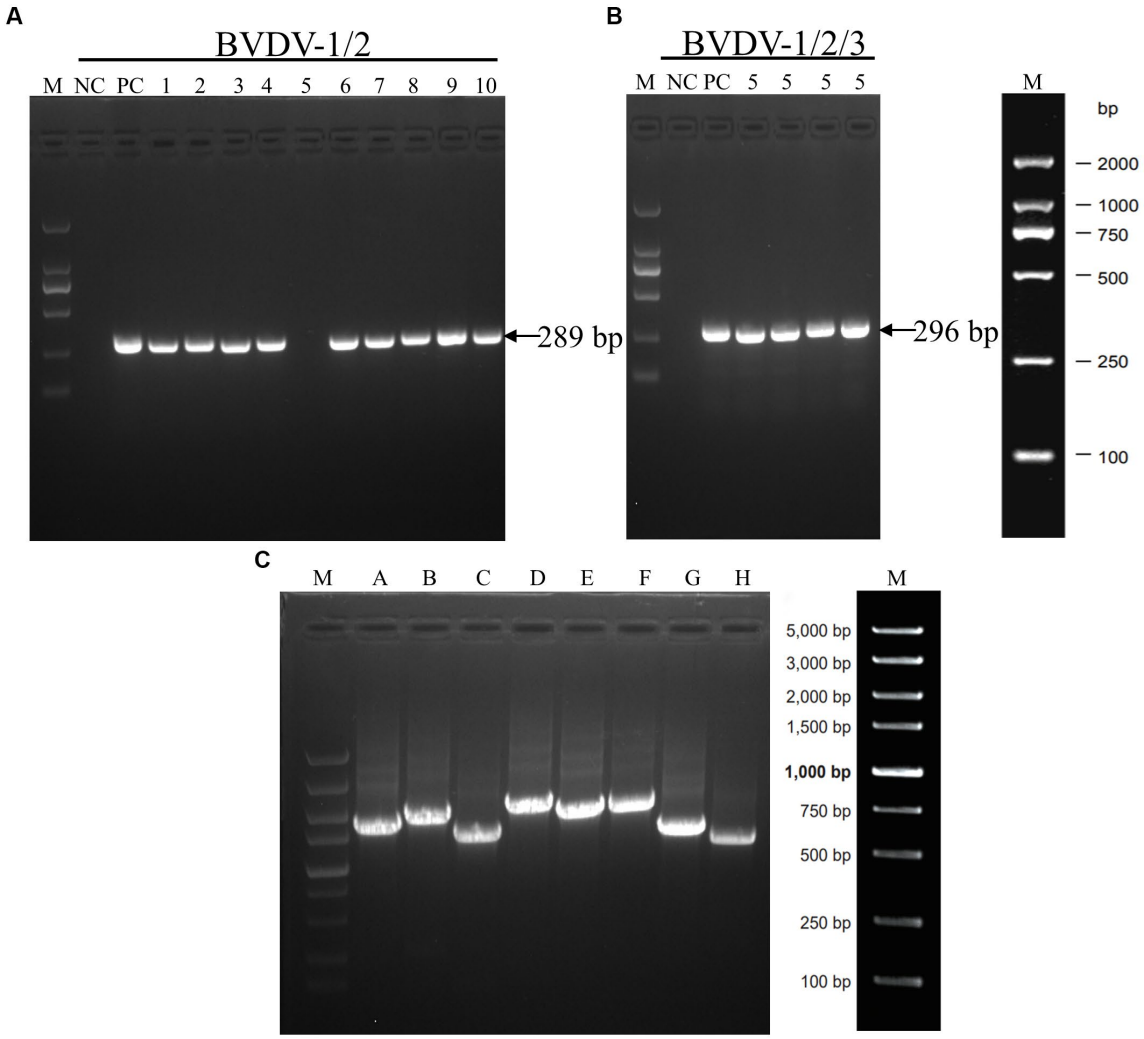
The results of RT-PCR tests conducted on some samples that tested positive for BVDV using ELISA. **(A)** The results of RT-PCR analysis of the BVDV-1/2 5’-UTR genomic fragment. Serum samples numbered 1 through 10 were analyzed. **(B)** RT-PCR analysis was performed on the BVDV1/2/3 5’-UTR genomic fragment using serum sample number 5. **(C)** The amplification of full-genome fragments using segmented primers **(A–H)**. The DNA marker is represented by the letter M, while negative and positive controls are indicated by NC and PC, respectively.

Ten positive samples were randomly selected for RT-PCR verification. The BVDV-1/2 primer was utilized for detection, and it was observed that all samples, except for sample No.5, exhibited the anticipated fragments (Figure 3A), implying that sample No.5 might not have been caused by BVDV-1/2 infection or could be a false positive. Therefore, further testing was conducted using the primers of BVDV-1/2/3, which revealed the presence of the expected fragment (Figure 3B), indicating that BVDV-3 might be the causative pathogen in sample No.5. To confirm this finding, full-length segmental amplification primers were designed for sample No.5, with reference to the complete sequence of BVDV-3 strain PB22487 (GenBank:KY762287.2). The results demonstrated the successful amplification of the target gene fragments *via* segmental amplification (Figure 3C). The RT-PCR results showed that the sample No.5 was infected with BVDV-3 strain.

### Sequencing and phylogenetic analysis

**Figure 4:**
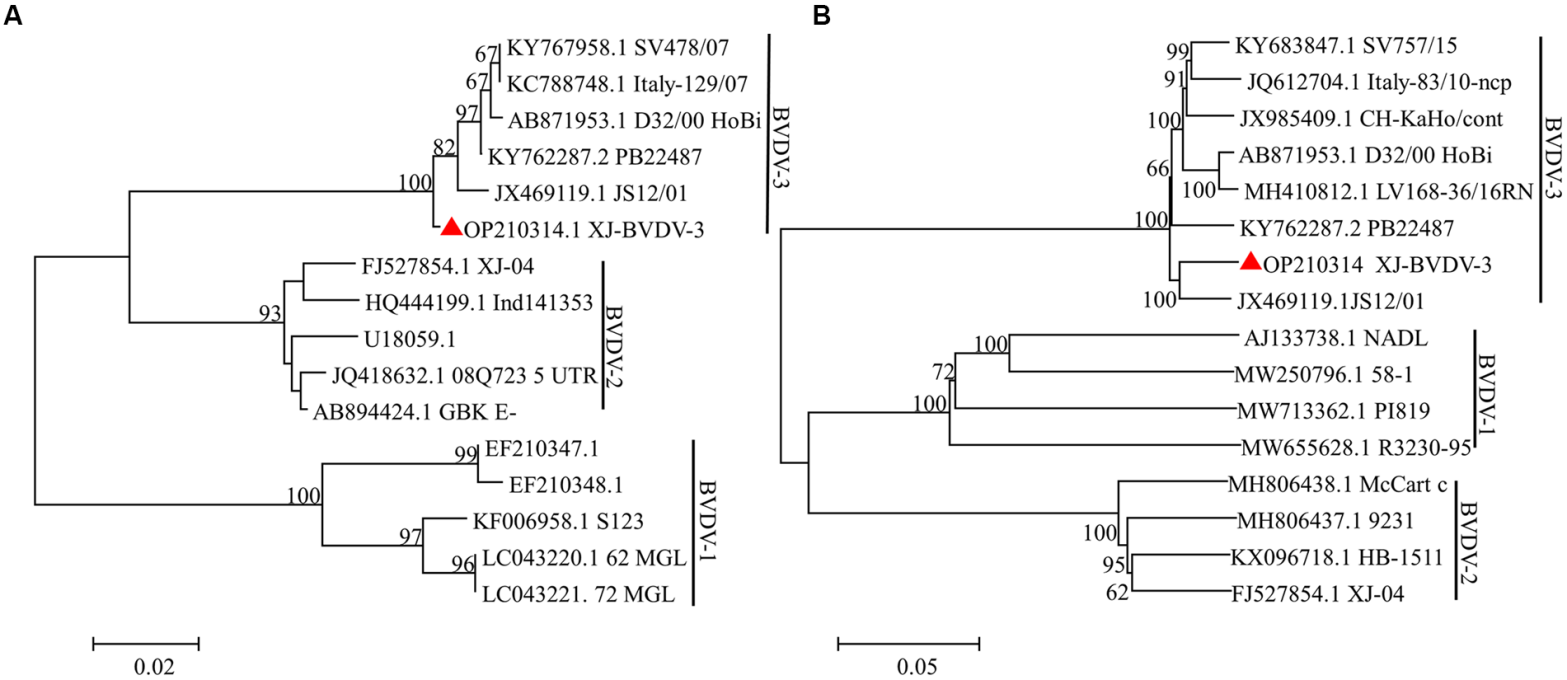
The phylogenetic trees based on the analysis of the sequences of the 5’-UTR and the whole genome. The trees were constructed using the neighbor-joining method and bootstrap testing. The numbers above the branches indicate the percentage of 1,000 bootstrap replicates that support each phylogenetic branch. **(A)** The phylogenetic trees based on the analysis of the sequences of the 5’-UTR. **(B)** The phylogenetic trees based on the analysis of the sequences of the whole genome. The red triangle represents the sequences identified in this study.

The aforementioned segments were ligated to the PMD-19-T vector and sent to Rui Biotech for sequencing. Three replicates were generated for each sample to ensure the accuracy of the results. Subsequently, the sequencing data were corrected and spliced using the DNAStar computer program package. The analysis revealed that the BVDV strain comprised of 12,239 nucleotides and 3,899 amino acids. To gain insights into the evolutionary relationships of the BVDV isolates, a phylogenetic analysis was performed using the MEGA 7.0 software. The whole-genome sequence was used to construct a phylogenetic tree using the neighbor-joining method. The results indicated that the BVDV strain clustered into BVDV-3 along with the JS12/01 strain (Figure 4). The newly identified BVDV strain was named XJ-BVDV-3. The whole-genome sequence information was uploaded to the National Center for Biotechnology Information (NCBI), and a GenBank login number (GenBank: OP210314) was obtained.

### Indirect immunofluorescence assay

**Figure 5:**
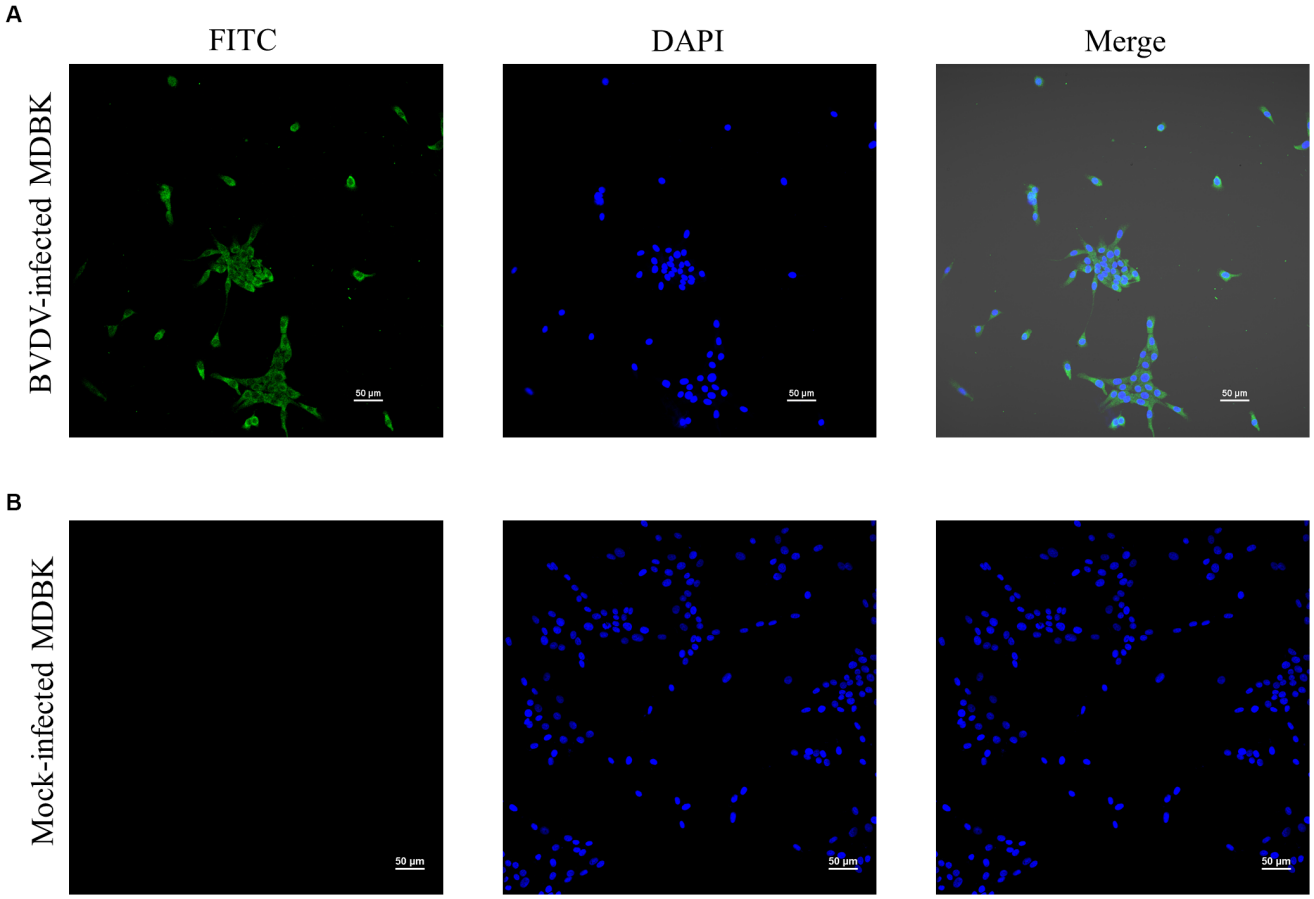
The identification of the virus isolate cultured in MDBK cells through IFA. **(A)** BVDV-infected MDBK cells. **(B)** Mock-infected MDBK cells.

MDBK cells still had no CPE when infected with P10 XJ-BVDV-3, indicating that XJ-BVDV-3 is a non-cytopathogenic BVDV. The IFA results showed that the MDBK cells infected with XJ-BVDV-3 showed green fluorescence, while the mock infected MDBK cells did not show green fluorescence (Figure 5).

## Discussion

BVDV has emerged as a global menace, wreaking havoc on animal health and production performance, and causing significant economic losses in the livestock industry worldwide (Ran et al., 2019). With the increasing demand for dairy and beef products, there has been a surge in investments in the cattle industry in many countries, including China, leading to frequent transportation of cattle and contributing to the rapid spread of BVDV across the country. A study by Deng et al. (2015) revealed that even clinically observed healthy cattle (such as cows, beef cattle, buffalo, and yaks) had a total positive rate of BVDV antibodies of 58.09%. Furthermore, other studies have reported that more than 46.7% of cattle farms in China tested positive for BVDV antigens, with a persistent infection rate of 2.2% in cattle herds, significantly higher than that of most Asian countries and even higher than that of European and American countries (Zhong et al., 2011; Guo, 2018; He et al., 2020).

At present, numerous subtypes of BVDV have been identified in China, including 1a, 1b, 1c, 1d, 1 m, 1o, 1p, 1q, 1u, 2a, 2b, as well as two novel subtypes, 1v and 1w, along with HoBiPeV (Deng et al., 2015; Yeşilbağ et al., 2017; Deng et al., 2020; Chen et al., 2021). BVDV-II is known to be more virulent, causing more severe clinical symptoms, and is frequently the culprit behind outbreaks in Europe and America (Nie et al., 2012; Zhao, 2015). HoBiPeV was first discovered in FBS originating from Brazil and subsequently found to infect cattle in China as well (Schirrmeier et al., 2004; Chen et al., 2021). In summary, there is a severe and complex BVDV epidemic in China with increasingly diverse genotypes, posing a significant threat to the sustainable development of the cattle industry. Consequently, appropriate measures should be taken to prevent and control this disease.

In this study, we procured 6,153 blood samples from 18 cattle farms, spanning 13 cities in four regions of Xinjiang (Figure 1; Table 1). Initially, we assayed the 6,153 serum samples for BVDV antibody. Results indicated that the antibody-positive rate was the lowest in the eastern region (19.46%), and the highest in the western region (84.99%; Figure 2B). This observation may be attributed to the fact that the western region had administered BVDV vaccines to its cattle 4 months prior to sampling. Furthermore, the serum antibody level of calves was significantly higher than that of adult cattle (Figure 2D), plausibly due to the presence of robust maternal antibodies in calves. The overall positive rate of BVDV antibody was found to be 53.68%, which is consistent with the prevalence rate of BVDV in dairy cattle reported in China (53.0%, Ran et al., 2019). Subsequently, we screened 588 serum samples for BVDV antigen. Interestingly, the antigen level of calves was significantly higher than that of adult cattle (Figure 2E), suggesting that calves are more susceptible to BVDV infection than adult cattle. As the western region has already been vaccinated, we did not test the antigen in this area. The positive rates of BVDV antigen in the remaining three regions, from low to high, were the eastern region (3.88%), southern region (4.08%), and northern region (7.24%), but no significant difference was observed among the three regions (Figure 2F). Due to the sampling site did not completely cover the entire Xinjiang region. Therefore, these data may not accurately reflect the prevalence of BVDV in various regions of Xinjiang.

We employed a randomized selection of 10 serum samples which tested positive for BVDV antigen by ELISA, for RT-PCR detection. As BVDV-3 had never been reported in Xinjiang, we initially used BVDV-1/2 primer for detection. The results showed that only the No.5 sample did not yield the expected band (Figure 3A). Subsequently, we utilized BVDV-1/2/3 primers to further test the No.5 sample, which generated the expected band at 296 bp, tentatively confirming that the No.5 sample was infected with BVDV-3 (Figure 3B). Because of the difficulty of virus isolation, most of the research focuses on the nucleic acid detection in 5’-UTR regions for virus identification and classification, which leads to the lack of reliable evidence for BVDV genotyping and comprehensive genetic characteristics analysis (Hou et al., 2019; Chang et al., 2021; Tian et al., 2021; Afify et al., 2022). To obtain conclusive results regarding the No.5 sample’s infection with BVDV-3, we designed full-length BVDV amplification primers with reference to the sequence of BVDV-3 strain PB22487 (GenBank: KY762287.2), and successfully amplified the target fragment in a segmented manner (Figure 3C). The resulting sequence was spliced and proofread using the DNAStar computer program package, which determined the full length of the virus genome as 12,239 nt. Comparison with the NCBI database confirmed that the No.5 sample was of the BVDV-3 type, designated as XJ-BVDV-3 (Figure 4), and the obtained data was uploaded to the NCBI database to obtain the login number: OP210314.1. In conclusion, this study confirmed the novel genotype BVDV-3 based on the phylogenetic analysis of the whole genome, indicating the reliability of identifying the novel genotype BVDV-3.

Since virus isolation is generally considered to be the gold standard for BVDV diagnosis (Saliki et al., 2000; Hou et al., 2019), we isolated and cultured the virus from sample No.5 using MDBK cells. We used IFA to detect the virus that was passed to the 10^th^ generation. Our results demonstrated that infected MDBK cells with XJ-BVDV-3 showed green fluorescence, while uninfected cells showed no fluorescence (Figure 5), indicating that we obtained a stable proliferating XJ-BVDV-3 viral strain in MDBK cells. Viral virulence is significance for understanding the pathogenic mechanism of viruses and selecting challenging strains to evaluate vaccines. However, considering the experimental cost, this study did not determine the pathogenic characteristics and virulence of XJ-BVDV-3, and it was still unknown whether it infects other animals besides cattle, such as pigs, sheep, etc. Since previous reports have shown that HoBiPeV can infect in cattle and small ruminants (Decaro et al., 2012; Bauermann et al., 2015; Bauermann and Ridpath, 2015). We can hypothesis that XJ-BVDV-3 strains can also infect pigs, sheep and other small ruminants, and further animal experiments are needed to verify the specific situation.

Our epidemiological investigation of BVDV in some areas of Xinjiang revealed a serious BVDV infection phenomenon in the region. During our investigation, we isolated the XJ-BVDV-3 strain for the first time. Phylogenetic and sequence analysis of the 5’-UTR gene and the whole genome revealed that the XJ-BVDV-3 strain belongs to the BVDV-3 genotype, indicating that the BVDV-3 strain is present in Xinjiang. In conclusion, real-time monitoring of the spreading of BVDV strains in cattle herds can provide reliable theoretical support for the development of diagnostic reagents and vaccines.

## Data availability statement

The original contributions presented in the study are included in the article/supplementary material, further inquiries can be directed to the corresponding authors.

## Author contributions

NY, MX, JS, and CC conceived and designed the project. NY, MX, ZM, HL, SS, XG, JL, ZY, HZ, HM, JY, YW, and ZW collected the samples. NY, MX, and ZM performed the experiments. NY, MX, and ZM performed the experiments. NY and MX analyzed and interpreted the data and drafted the initial manuscript. JS and CC reviewed and critically revised the initial draft. All authors contributed to the article and approved the submitted version.

## Funding

The authors declare that this study received funding from the Transformation and Application Demonstration of Rapid Screening Technology Achievements for Important Animal Diseases in Intensive Breeding (grant no. 21322912D) and The Special Fund for Science and Technology Development of Xinjiang Production and Construction Corps “Research and Application of Rapid Diagnosis Kit for Bovine Viral Diarrhea/ Mucosal Disease (BVDV/MD)” (grant no. 2017BA044). The funder was not involved in the study design, collection, analysis, interpretation of data, the writing of this article or the decision to submit it for publication.

## Conflict of interest

The authors declare that the research was conducted in the absence of any commercial or financial relationships that may be construed as a potential conflict of interest.

## Publisher’s note

All claims expressed in this article are solely those of the authors and do not necessarily represent those of their affiliated organizations, or those of the publisher, the editors and the reviewers. Any product that may be evaluated in this article, or claim that may be made by its manufacturer, is not guaranteed or endorsed by the publisher.
